# Inaccurate self-report of olfactory dysfunction in REM Sleep Behaviour Disorder and implications for prognosis

**DOI:** 10.1016/j.prdoa.2022.100176

**Published:** 2022-12-17

**Authors:** Amber Roguski, Michal Rolinski, Matt W. Jones, Alan Whone

**Affiliations:** a School of Physiology, Pharmacology and Neuroscience, University of Bristol, Bristol, United Kingdom; b Department of Neurology, Torbay Hospital, Torquay, United Kingdom; c Department of Neurology, Southmead Hospital, Bristol, United Kingdom; d Translational Health Sciences, University of Bristol, Bristol, United Kingdom

**Keywords:** Parkinson’s disease, REM Sleep Behaviour Disorder (RBD), Prodromal, Prognosis, Biomarker, Olfaction

## Abstract

**Introduction:**

The earliest stages of alpha-synucleinopathies are accompanied by non-specific prodromal symptoms such as diminished sense of smell, constipation and depression, as well as more specific prodromal conditions including REM Sleep Behaviour Disorder (RBD). While the majority of RBD patients will develop an alpha-synucleinopathy, one of the greatest clinical challenges is determining whether and when individual patients will phenoconvert. Clinical evaluation of a patient presenting with RBD should therefore include robust and objective assessments of known alpha-synucleinopathy prodromes.

**Methods:**

This study compared olfactory function self-report measures with psychophysical ‘Sniffin’ Stick 16-item Identification’ test scores in Control (n = 19), RBD (n = 16) and PD (n = 17) participants.

**Results:**

We confirm that olfactory test scores are significantly diminished in RBD and PD groups compared to Controls (p < 0.001, One-Way ANOVA with Tukey-Kramer Post-Hoc, effect size = 0.401). However, RBD participants were only 56 % accurate when self-reporting olfactory dysfunction, hence markedly less likely to perceive or acknowledge their own hyposmia compared to Controls (p = 0.045, Fisher’s Exact Test, effect-size = 0.35).

**Conclusion:**

When isolated RBD presents with hyposmia, there is an increased likelihood of phenoconversion to Parkinson’s Disease (PD) or Dementia with Lewy Bodies (DLB); unawareness of olfactory dysfunction in an individual with isolated RBD may therefore confound differential diagnosis and prognosis. Our results evidence the fallibility of olfactory function self-report in the context of RBD prognosis, indicating that clinical assessments of RBD patients should include more reliable measures of olfactory status.

## Highlights

• REM Sleep Behaviour Disorder (RBD) concomitant with olfactory dysfunction is indicative of underlying synucleinopathy such as Parkinson’s Disease (PD) and Dementia with Lewy Bodies.
• Clinicians often rely on olfactory function self-report from RBD patients.
• Accuracy of olfactory function self-report was compared to Sniffin’ Sticks 16-item olfactory function test scores in Control, PD and RBD groups.
• RBD group were less accurate in their self-report of olfactory dysfunction compared to Control and PD groups.
• Olfactory function self-report may not be suitable in an RBD population context and may reduce prognosis accuracy.

## 1 Introduction

The alpha-synucleinopathies Parkinson’s disease (PD), Dementia with Lewy Bodies (DLB) and Multiple System Atrophy (MSA) are a group of neurodegenerative disorders which, despite shared pathology of misfolded alpha-synuclein protein aggregation, have varying symptom profiles. The earliest stages of alpha-synucleinopathy neurodegeneration are accompanied by non-specific prodromal symptoms such as diminished sense of smell, constipation and depression, as well as more specific prodromal symptoms such as REM Sleep Behaviour Disorder (RBD) [1], [2], [3]. The combination of prodromal symptoms and extent of their severity can be indicative of which alpha-synucleinopathy is developing [4], [5], [6].

The most specific prodrome for alpha-synucleinopathies is isolated RBD, where the condition is not caused by a known mechanism such as medication, pathological stress or specific lesion [7]. Prevalence of hyposmia (diminished sense of smell) and anosmia (total loss of sense of smell) has been consistently shown to be higher in isolated RBD patients compared to controls [8], [9]. When isolated RBD presents with hyposmia, this is indicative of an underlying synucleinopathy and there is an increased likelihood of phenoconversion to PD or DLB [10]. In contrast, MSA is associated with relatively preserved olfactory function [10], [11], [12]. Hyposmia may therefore serve as a differentiating prodrome of a-synucleinopathies and prognostic predictor when co-occurring with isolated RBD.

There is conflicting evidence as to whether olfactory function progressively deteriorates over the course of alpha-synucleinopathy development, with different domains demonstrating different progression profiles. Longitudinal studies utilising odour identification paradigms (e.g. Sniffin’ Sticks identification subtest; UPSIT-40) found olfaction does not progressively decline in prodromal RBD cohorts, and instead is a stable and prognostic indicator of synucleinopathy [10], [13]. This stable RBD olfactory profile potentially reflects a floor-effect, wherein the alpha-synuclein pathology responsible for olfactory dysfunction (olfactory bulb, anterior olfactory nucleus, orbitofrontal cortex) has already maximally occurred in the early prodromal stage [10], [13]. However, other olfaction domains (threshold detection, odour discrimination) have been shown to decline in prodromal RBD cohorts over the course of 4 years [13].

Self-report of olfactory dysfunction is unreliable and declines with age, with poor sensitivity ranging in reports from 12 to 35 % [14], [15], [16], [17]. However, when isolated RBD patients are clinically assessed, the majority of clinicians rely on patient self-reports and do not perform further olfaction assessments. Unawareness of olfactory dysfunction in an individual with isolated RBD may impact differential diagnosis and prognosis.

Here, we therefore test whether there is a mismatch between the self-reported olfactory function and clinically-assessed olfaction test scores in RBD and PD patients compared to controls.

## 2 Methods

### 2.1 Participants

103 potential participants were contacted and screened for inclusion. 54 people met the study inclusion criteria and verbally agreed to participate; 2 participants subsequently withdrew from the study prior to enrolment due to the time commitment required. 52 participants gave written consent to participate and were enrolled onto the study. Data from *n* = 52 participants are reported here.

Patient group participants (RBD and PD groups) were recruited from the Neurology and Neuropsychiatry services of Southmead Hospital, North Bristol NHS Trust, UK. Control participants (*n* = 19) were recruited from the local population, including partners or friends of patient group participants.

Study inclusion criteria required PD participants to have received their diagnosis in the previous 3 years, meaning they were in the relatively early stages of Parkinson’s. Of the *n* = 17 PD participants reported in this study, 6 had ‘probable-RBD’ (p-RBD) and were categorised as such using the criteria of a history of dream enactment or a score ≥ 6 on the REM Sleep Disorder Screening Questionnaire (RBDSQ) [18]. None of the PD participants with p-RBD had polysomnography-confirmed RBD, and for most p-RBD participants their RBD symptoms developed at the same time as their PD diagnosis.

All RBD (*n* = 16) participants had a polysomnography-confirmed RBD diagnosis according to ICSD criteria (depending on date of diagnosis, either ICSD-2 [19] or ICSD-3 [20] criteria) and were considered by their clinician to have ‘isolated’ RBD rather than due to a secondary cause.

The study protocol was approved by the University of Bristol’s Research Ethics Department (RED), the South West-Central Bristol NHS Research Ethics Committee (REC) and the Health Research Authority (HRA). REC reference: 19/SW/0103. All participants provided written informed consent following a full discussion of the study procedure.

### 2.2 Olfaction assessment

Olfactory function was quantified using the 16-item Sniffin’ Sticks Smell Test (Burghart GmBh, Wedel, Germany). This test assesses odour identification using a forced-choice paradigm, wherein an odorant is presented to the participant for 2–3 s and they must identify the scent out of a choice of 4 options. A 30 s pause between odours minimises odour cross-contamination. As such, the duration of the test is approximately 12 min. Participants can score between 0 (no correct identification) and 16 (all odorants identified correctly).

As part of the Sniffin’ Sticks smell test, participants report any recent cold or flu, whether they are aware of any olfactory or gustatory changes and the duration of these changes. Although the Sniffin’ Sticks smell test primarily interrogates olfaction, participants are questioned on their sense of taste due to the strong connection between olfactory and gustatory function. Participants who reported olfactory dysfunction were classified as having ‘perceived hyposmia/anosmia’, while those participants who reported gustatory dysfunction were classified as having ‘perceived ageusia’. Participants are also asked to report their first language and whether they speak other languages fluently, due to the cultural context of sensory assessments.

There are several criteria available to classify olfactory function as normosmic, hyposmic or anosmic using Sniffin’ Sticks [21], [22], [23], including cut-off criteria provided in the test kit (Burghart Messtechnik GmBH). This study utilises Oleszkiewicz et al’s age-adjusted percentile scores [23] (Supplementary Table 1) to classify olfactory function.

**Table 1:** Demographics and Relevant Medical History results. All continuous variable results are presented as mean and standard deviation (s.d.) unless otherwise specified. All categorical variable results are presented as frequency (n) and percentage (%) unless otherwise specified. For p-values & statistical tests, a = One-Way ANOVA with Tukey post-hoc, b = Kruskal Wallis, c = Fisher’s Exact Test with Bonferroni correction d = Pearson’s Chi-Square Test for Independence with Bonferroni Correction.

	Control (n = 19) ◊	RBD (n = 16) †	PD (n = 17) ‡	p-value; post-hoc	Test Statistic	Effect Size
**Sex**				ns (0.637)c	1.109 (2)	0.14
Male	14 (73.7 %)	14 (87.5 %)	13 (76.5 %)			
Female	5 (26.3 %)	2 (12.5 %)	4 (23.5 %)			

**Age**	69.57 (8.77)	64.64 (9.05)	66.73 (9.3)	ns (0.277)a	*F*(2,49) = 1.319	0.05
Male	68.87 (9.98)	64.46 (7.07)	66.09 (7.84)	ns (0.383)a	*F*(2,38) = 0.985	0.05
Female	71.55 (3.99)	65.91 (23.97)	68.81 (14.43)	ns (0.860)a	*F*(2,8) = 0.154	0.04

Duration of RBD Symptoms (years ± s.d)	–	8.75 (7.17)	–	–	–	–

Duration of RBD Diagnosis (years ± s.d)	–	2.8 (1.37)	–	–	–	–

Duration of PD motor Symptoms (years ± s.d)	–	–	5.88 (6.77)	–	–	–

Duration of PD Diagnosis (years ± s.d)	–	–	1.82 (0.882)	–	–	–

Diagnosed Nasal Pathology	2 (10.52)	2 (12.5)	0 (0)	ns (0.446)c	2.175 (2)	0.2

**Smoking Status**				ns (0.77)c	0.628 (2)	0.11
Never Smoked	13 (68.4)	9 (56.3)	11 (64.7)			
Ex-Smoker	6 (31.6)	7 (43.8)	6 (35.3)			
Smoker	0 (0)	0 (0)	0 (0)			
Occupational Exposure to Pollutants	2 (10.5)	4 (25)	4 (23.5)	ns (0.495)c	1.694 (2)	0.18

Agreement between self-reported and clinically-assessed olfactory function was defined as follows:

**Correctly Perceived Olfaction** – individuals’ perception of their sense of smell matches their Sniffin’ Sticks score.

**Incorrectly Perceived Normosmia** – individuals perceive their sense of smell to be within the normosmic range, but their Sniffin’ Sticks score is hyposmic or anosmic.

**Incorrectly Perceived Hyposmia /Anosmia** – individuals perceive their sense of smell to be within the hyposmic /anosmic range, but their Sniffin’ Sticks score is normosmic.

### 2.3 Data analysis

Data were analysed using SPSS Version 26 software (SPSS Inc., Chicago, Ill., USA). Shapiro-Wilk tests for normality were followed by 2-sided parametric or non-parametric tests, chosen as appropriate to calculate inferential statistics. Corrections for multiple comparisons were used for post-hoc analyses. Effect size is given as Cramer’s V for Fisher’s test and Eta Squared for ANOVA & Kruskal Wallis analyses.

Accuracy, sensitivity and specificity values were calculated as described in [24].

## 3 Results

### 3.1 Demographics

All participants were living in the South-West of England and the majority were of White British ethnicity (*n* = 47, 90.4 %). The remaining participants identified with White European ethnic backgrounds or dual heritage backgrounds. 96.1 % (*n* = 50) of participants were native speakers of English, while the remaining participants spoke fluent English. The majority of participants were male (*n* = 41, 78.85 %).

There were no significant differences in the age or sex distributions between the 3 participant groups. In all groups, female participants were slightly older than their male counterparts, though not significantly so (Two-Way Between Groups ANOVA *F*(2,46) = 0.013, p = 0.987, partial eta squared = 0.001).

In addition to the results shown in Table 1, there were no significant differences between groups when measures of socioeconomic status (accommodation status, vehicle access, employment status & position) were analysed.

There were no significant differences between groups in relevant medical history. Control and RBD groups contained a low number of participants with diagnosed nasal pathologies, specifically nasal polyps and rhinitis. Health status predictors (years of education, marital status) were also similar across groups.

### 3.2 Self-report olfactory and gustatory function

**Fig. 1:**
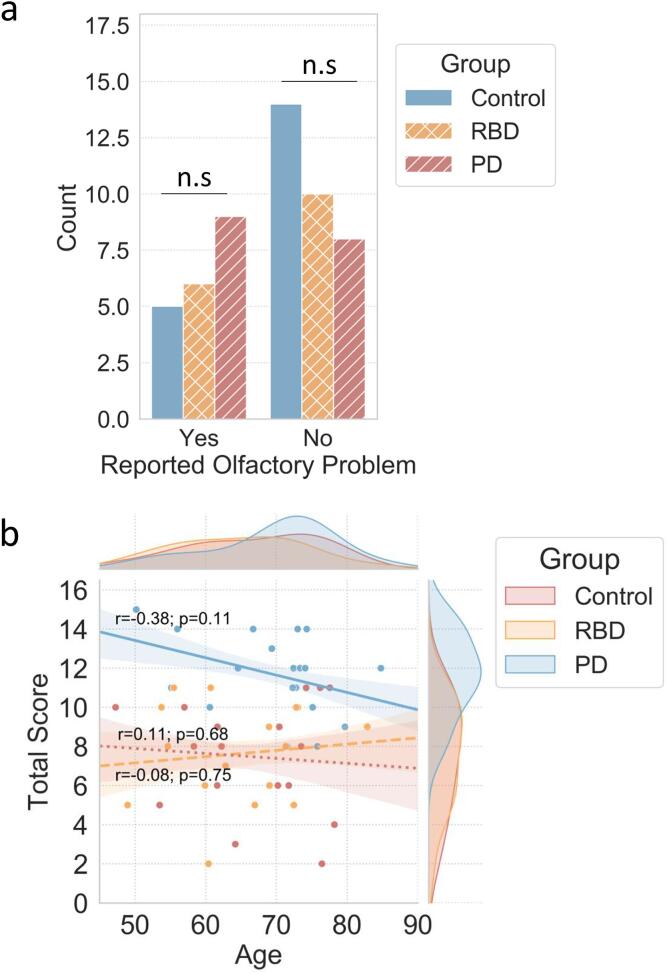
PD and RBD participants are more likely to incorrectly perceive normal olfactory function: (a) *Self-reported olfactory problem frequencies per group. Differences between group were not significant (Fisher’s exact test p = 0.272).* (b) *Scatter plot showing the distributions of Age and Sniffin’ Sticks score according to age. Marginal axes show respective density curves for the distribution of ages (x-axis) and Total Sniffin’ Sticks score (y-axis) for each group. Line of best fit (±SE) and Pearson’s correlation coefficient is calculated for each group to describe the relationship between age and olfactory function (Control r = -0.38, p = 0.11; RBD r = 0.11, p = 0.68; PD r = -0.08,p = 0.75).*

Self-reported olfactory dysfunction, or perceived hyposmia, was not significantly different between the groups. Of the 3 groups, only the PD group included a majority of participants self-reporting a diminished sense of smell (Table 2; Fig. 1a).

**Table 2:** Olfactory Perception, Sniffin’ Sticks Smell Test Results and Olfaction Categorisation. All continuous variable results are presented as mean and standard deviation (s.d.) unless otherwise specified. All categorical variable results are presented as frequency (n) and percentage (%) unless otherwise specified. For p-values & statistical tests, a = One-Way ANOVA with Tukey post-hoc, b = Kruskal Wallis, c = Fisher’s Exact Test with Bonferroni correction d = Pearson’s Chi-Square Test for Independence with Bonferroni Correction.

	Control (n = 19) ◊	RBD (n = 16) †	PD (n = 17) ‡	p-value; post-hoc	Test Statistic	Effect Size
Perceived Hyposmia/Anosmia (*n*, %)	5 (26.3 %)	6 (37.5 %)	9 (52.9 %)	ns (0.272)c	2.646 (2)	0.228

Perceived Hyposmia/Anosmia Duration (years ± s.d)	30.3 (20.69)	12.33 (5.72)	12 (15.23)	ns (0.144)b	*X*^2^(2,20) = 3.88	−0.007

Perceived Ageusia (*n*, %)	2 (10.5 %)	4 (25 %)	5 (29.4 %)	ns (0.33)c	2.199	0.202

Perceived Ageusia Duration (years ± s.d)	15.2 (20.86)	10 (0)	14.4 (20.11)	ns (0.729)b	*X*^2^(2,11) = 0.633	−0.42

Sniffin’ Sticks Score (±s.d)	11.68 (2.1)	7.63 (2.6)	7.47 (2.85)	<0.001a;	*F*(2,49) = 16.440	0.401
				◊>† <0.001,		
				◊>‡ <0.001		

**Olfaction Categorisation** (*n*, %)				<0.001c;	n/a	0.442
Normosmic	17 (89.5 %)	5 (31.3 %)	4 (23.5 %)	◊> †,‡ 0.001		
Hyposmic	2 (10.5 %)	3 (18.8 %)	5 (29.4 %)	ns		0.501
Anosmic	0 (0 %)	8 (50 %)	8 (47.1 %)	◊< †,‡ 0.001		

**Olfaction Perception** (*n*, %)						
Correctly Perceived Olfaction	14 (73.68 %)	9 (56.25 %)	11 (64.71 %)	ns (0.544)c	n/a	0.3
Incorrectly Perceived Normosmia	1 (5.26 %)	6 (37.5 %)	5 (29.41 %)	0.045c;	n/a	0.35
Incorrectly Perceived Hyposmia	4 (21.05 %)	1 (6.25 %)	1 (5.88 %)	ns(◊<†,‡ 0.06)	n/a	0.185

A minority of participants in all groups self-reported gustatory dysfunction, or perceived ageusia. Duration of perceived hyposmia or ageusia was not significantly different between groups.

### 3.3 Sniffin’ Sticks smell test

RBD and PD groups scored significantly lower than Controls on the Sniffin’ Sticks smell test (Table 2; Fig. 1b). There was no significant difference between the RBD and PD mean Sniffin’ Sticks scores, indicating similar levels of olfactory dysfunction between the two groups. When the PD group was stratified into p-RBD PD and PD *without* p-RBD, there was no significant difference between mean total Sniffin’ Sticks scores (p-RBD PD mean score = 7.5 ± s.d. 2.59; PD *without* p-RBD mean score = 7.45 ± s.d. 3.11; Independent Samples T-Test *t*(15) = 0.03, p = 0.98, two-tailed, eta squared = 0.015).

The majority (89.5 %) of Control participants were categorised as ‘normosmic’. The majority of RBD and PD participants experienced olfactory dysfunction of some degree, and both RBD and PD groups had a significantly greater proportion of ‘anosmic’ individuals compared to Controls (p < 0.001, Fisher's Exact Test).

There was no difference in Sniffin’ Sticks score between individuals diagnosed with nasal pathology (*n* = 4; mean score = 10.00 ± s.d. 0.82) and those without nasal pathology (*n* = 48; mean score = 8.98 ± s.d. 3.23). Secondary analyses were conducted after removing individuals with nasal pathology, however this did not impact the overall results. Similarly, there was no significant difference in Sniffin’ Sticks score between non-smokers (*n* = 33; mean score = 8.79 ± s.d 3.2) and ex-smokers (*n* = 19; mean score = 9.53 ± s.d. 3.17) (independent samples *t*-test; t(50) = -0.8, p = 0.43, two-tailed). The magnitude of the differences in the means was of small effect (Cohen’s d = 0.23), suggesting limited association between variables.

### 3.4 Mismatch between self-report and Sniffin’ sticks olfactory categorisation

The majority of participants had a correct perception of their olfactory function (Table 2). However, there was a significant difference in the occurrence of Incorrectly Perceived Normosmia across groups (p = 0.045, Fisher’s Exact Test): RBD and PD groups had more participants self-reporting normosmia while scoring in the hyposmic or anosmic range on the Sniffin’ Sticks smell test. Subgroup analysis of p-RBD PD and PD *without* p-RBD found no clear differences in olfaction perception between groups (correctly perceived normosmia, incorrectly perceived normosmia and incorrectly perceived hyposmia; Fisher’s exact test two-tailed p = 1.0 for all analyses). The RBD group contained the greatest number of participants with incorrectly perceived normosmia; 37.5 % compared to 5.3 % Control.

The discrepancies between olfaction categorisation and self-reported olfactory function (Table 2; Supplementary Table 2) were explored by calculating the accuracy, sensitivity and specificity of the respective groups’ self-report (Table 3), using the 16-item Sniffin’ Sticks test as a gold-standard psychophysical assessment of olfactory function.

**Table 3:** Accuracy, sensitivity and specificity of olfactory function self-report.

Group	Accuracy	Sensitivity	Specificity
Control	73.68 %	50 %	76.47 %
RBD	56.25 %	45.45 %	66.67 %
PD	64.71 %	61.54 %	75 %

For control participants, self-report was 74 % accurate, compared to 56 % and 65 % for RBD and PD respectively. Sensitivity and specificity values were lowest for the RBD group (45 % and 67 % respectively) compared to the Control and PD groups.

## 4 Discussion

This study compared olfactory function self-perceptions with psychophysical assessment scores in Control, RBD and PD participants with similar age, sex, socioeconomic status and health profile. We found a significant difference between groups when comparing the number of participants who incorrectly perceived their sense of smell to be normosmic. Specifically, RBD participants were more likely to incorrectly perceive their sense of smell to be normosmic when compared to Controls, and to a lesser degree when compared to the PD group.

Given that self-report is the most common way in the UK for clinicians to assess patient olfaction, we sought to investigate whether there was a mismatch between participant’s perceptions of their sense of smell compared to their Sniffin’ Sticks score. We hypothesised that RBD patients may be unaware of the association between olfactory impairment, RBD and PD and therefore more likely to be unaware of their own sense of smell. If RBD patients do not report olfactory changes to clinicians, this may have impacts for their healthcare and the clinicians’ ability to provide an accurate prognosis given that ofactory disturbance associates with the rate of RBD phenoconversion and that ofactory disturbance is less common in MSA [10], [11], [12].

We found that self-report was less accurate than psychophysical olfactory testing in the RBD group compared to Control and PD groups, with lower sensitivity and specificity. Our results indicate that RBD patients are less likely to be aware of any olfactory dysfunction than Control and PD individuals, calling into question the reliance of clinicians on olfactory self-report for RBD prognosis, where olfactory dysfuction arising in RBD increases the likelihood of phenoconversion.

Pathologies which cause hyposmia range from transient inflammation to neuronal death. Diminished olfactory function is therefore a non-specific symptom of many conditions, from short-term illness including COVID-19 [25] to long-term conditions such as chronic rhinosinusitis and traumatic brain injury [26], [27].

The distinction between non-specific and prognostic olfactory dysfunction was investigated by considering factors which may influence olfaction such as smoking history, diagnosed nasal pathologies and exposure to pollutants. No participants in the study were current smokers, and the majority of participants in each group had never smoked. There were no significant differences in the number of ex-smokers between the 3 groups. Additionally, while current smoking is associated with olfactory dysfunction, function is rescued by smoking cessation [28]. In both the Control and RBD groups, *n* = 2 participants reported diagnosed nasal pathology, specifically rhinitis or nasal polyps. Neither smoking history nor nasal pathology significantly impacted on Sniffin’ Sticks score. There was no significant difference in the number of participants per group who had exposure to pollutants, which ranged from fertiliser and carbon dust to petrol vapours. The low prevalence of these exposures, coupled with the limited epidemiological literature [29], make it difficult to comment on the impact of these exposures on olfaction in the current study.

Across the 3 groups, the majority of participants did not report any olfactory or gustatory dysfunction. The PD group had a non-significantly greater number of participants reporting olfactory dysfunction than RBD and Control groups (PD 52.9 %; RBD 37.5 %, Control 26.3 %). This perhaps reflects the simultaneous and juxtaposing phenomena that while PD patients are aware of the association between olfactory impairment and PD, they still over-rate their sense of smell compared to controls [30]. The interpretation of these results would benefit from a larger sample size to explore the mechanisms and psychology of this phenomenon.

When participants’ olfaction was tested using the Sniffin’ Sticks 16-item identification smell test, RBD and PD group scores were significantly lower than Controls, with similar performance in RBD and PD groups (RBD mean ± SD 7.63 (2.6); PD mean ± SD 7.47 (2.85) p = 0.983). When participants’ olfactory function was categorised as ‘normosmic’, ‘hyposmic’ or ‘anosmic’ according to age-adjusted criteria applied to Sniffin’ Sticks score [19], significantly more RBD and PD participants were ‘anosmic’ compared to Controls. All 3 groups contained similar numbers of ‘hyposmic’ participants, likely due to the age of participants and increased incidence of health conditions [16], [31].

The Sniffin’ Sticks scores and categorisations support the literature that isolated RBD is associated with diminished sense of smell to an extent comparable with PD olfactory profiles [32]. Additionally, the similar scores between the patient groups are consistent with olfactory (dys)function not progressively worsening over time, but instead plateauing at some early prodromal stage of the PD neurodegenerative process, with early progression occurring before reaching a floor effect [33].

Finally, we did not find any differences in the Sniffin’ Sticks scores nor in olfaction perception when we conducted p-RBD PD and PD without p-RBD subgroup analysis, suggesting the extent of olfactory dysfunction and awareness of it is independent of concomitant RBD or PD subtypes. We therefore interpret these preliminary findings as a cognitive bias, in that people with isolated RBD are not always aware that olfactory dysfunction is commonly associated with RBD. Olfactory dysfunction is a far more recognised symptom of PD and thus we propose people with PD are more likely to be aware of a deficit.

Our findings warrant further investigation into the relevance of olfaction self-report within the clinic when considering RBD prognosis. Future work should build upon this by applying the Extended Sniffin’ Sticks test to contextualise our findings within the larger Threshold, Discrimination and Identification (TDI) composite score, which additionally assesses odour threshold detection and odour discrimination [34]. Replication of this work using other commonly-used psychophysical olfaction assessments, such as the University of Pennsylvania Smell Identification Test (UPSIT), would also be useful.

### 4.1 Study limitations

The core hypothesis motivating this paper arose from incidental observations during the data collection stage of a broader study of RBD, PD and control groups. Thus, the data analysed in this paper were not explicitly collected to test the hypothesis.

The participant sample size is relatively small and this may contribute to the conclusions drawn. While the majority of the associations reported are reinforced by effect size (small effect size for non-significant results, large effect size for significant differences), we report several variables where effect size is ‘medium’ – namely whether participants correctly perceive their olfactory function (non-significant difference between groups) or if they incorrectly perceive normosmia (significant difference between groups). The interpretation of these results may benefit from a larger sample size.

Another limitation of this study is the over-representation of the white, older male identity within our cohort. While this is common practice within biomedical research – particularly when focusing on age-related neurodegeneration – it has countless damaging impacts upon science, society and healthcare, ranging from the generalisability of results to reinforcing marginalisation of particular identities. We acknowledge that measures to recruit participants with diverse identities were not sufficient within this study.

## 5 Conclusion

To the best of our knowledge, this is the first paper to consider the fallibility of olfactory function self-report in the context of RBD prognosis. This work puts forward the case for routine objective testing of olfactory function in the clinic upon initial RBD diagnosis as a cost-effective and accessible method to inform prognosis and potentially improve healthcare outcomes for RBD patients.

## Funding Disclosures and Conflict of Interest

*Funding Sources and Conflict of Interest*

AR is supported by a UKRI/BBSRC CASE PhD studentship in partnership with Eli Lilly & Company [Grant No BB/S507295/1]. There is no conflict of interest.

*Financial Disclosures for the previous 12 months*

MWJ is supported by a Wellcome Trust Senior Fellowship in Basic Biomedical Sciences and has no additional disclosures to report. There is no conflict of interest.

AW is salaried by the University of Bristol. In the past 12 months AW has received speaker fee honorariums from Novo Nordisk and has advised Vivifi Biotech for a total of 10 h. AW has received research grants from North Bristol Trust Charities; University of Bristol Alumni Scobie Award; Cure Parkinson’s Trust; EPSRC; Wellcome Trust; Academy of Medical Sciences; Elizabeth Blackwell Institute for Health Research; David Telling Trust; NIHR. There is no conflict of interest.

## Declaration of Competing Interest

The authors declare that they have no known competing financial interests or personal relationships that could have appeared to influence the work reported in this paper.
