# Supplementary material for: Inaccurate self-report of olfactory dysfunction in REM Sleep Behaviour Disorder and implications for prognosis
Source: Clin Park Relat Disord. 2022 Dec 17;8:100176. doi: 10.1016/j.prdoa.2022.100176 (PMC9804136; doi:10.1016/j.prdoa.2022.100176)
Supplement: Supplementary data 1 [file mmc1.docx]

Supplementary Figures

|  | Normosmia | Hyposmia (10^th^ percentile) | Anosmia (5^th^ percentile) |
| --- | --- | --- | --- |
| 41-50 years | >=12 | 11 | 10 |
| 51-60 | >=12 | 11 | 9 |
| 61-70 | >=11 | 10 | 7.25 |
| 71-80 | >=8 | 7 | 5 |
| 81+ | >=5 | 4 | 2.2 |

Supplementary Table 1: *Age-adjusted olfactory function criteria, based on percentile scores from* (Oleszkiewicz et al., 2019)*.*

|  | | Sniffin’ Sticks Score Categorisation | |
| --- | --- | --- | --- |
|  |  | **Normosmic**  Control *n*=17  RBD *n*=5  PD *n*=4 | **Hyposmic/Anosmic**  Control *n*=2  RBD *n*=11  PD *n*=13 |
| Self-reported Olfaction Problems | **Yes**  Control *n*=5  RBD *n*=6  PD *n*=9 | Control = 4  RBD = 1  PD = 1 | Control = 1  RBD = 5  PD = 8 |
|  | **No**  Control *n*=14  RBD *n*=10  PD *n*=8 | Control = 13  RBD = 4  PD = 3 | Control = 1  RBD = 6  PD = 5 |

Supplementary Table 2: *Frequency table to explore relationship between self-reported olfactory problems and olfaction category determined using the Sniffin’ Sticks test.*
